# Identification of Personal Factors in Motor Neurone Disease: A Pilot Study

**DOI:** 10.1155/2011/871237

**Published:** 2011-07-07

**Authors:** Louisa Ng, Fary Khan

**Affiliations:** Department of Medicine, Dentistry and Health Sciences, The University of Melbourne, Royal Melbourne Hospital, Poplar Road, Parkville, Melbourne, VIC 3052, Australia

## Abstract

Motor neurone disease (MND) is a devastating condition. This preliminary study aims to identify relevant personal factors affecting the experience of living with MND from the perspective of persons with MND (pwMND) in an Australian cohort. A prospective cross-sectional survey of pwMND (*n* = 44) using an open-ended questionnaire identified personal factors that were categorised thematically. Standardised questionnaires assessed disease severity: depression, anxiety, and stress and coping strategies. Personal factors identified included demographic factors (socioeconomic status), emotional states (depression, anxiety, and fear), coping strategies (problem-based coping and denial), personality, beliefs (religious and personal values), attitudes (of the patient), and others (such as perceived support). An understanding of personal factors by treating clinicians is essential in the provision of optimal care in MND. This study may assist in the development of personal factors within the International Classification of Functioning, Disability, and Health for improved consensus of care and communication amongst treating clinicians.

## 1. Introduction

Motor neurone disease (MND) (amyotrophic lateral sclerosis) is a relatively rare neurodegenerative disorder of the motor system in adults characterized by the loss of motor neurons in the cortex, brain stem, and spinal cord, manifested by upper and lower motor neuron signs and symptoms affecting bulbar, limb, and respiratory muscles. Death usually occurs three-to-five years after onset from respiratory failure, but some may survive for a decade or more [1]. The burden of disease and economic impact of MND upon patients, their caregivers (often family members), and on society is substantial. It often begins long before the actual diagnosis is made and increases with increasing disability and the need for medical equipment and assisted care [2]. 

With no cure currently available, the challenge in MND is to prolong independence, prevent complications, and optimise quality of life (QoL). This is best met by a multidisciplinary team with a focus on symptomatic, rehabilitative, and palliative care [3, 4], through holistic interventions (incorporate personal and environmental factors) that span the spectrum of the disease. A significant part of the palliative rehabilitation process is the self-empowerment of patients and their families and helping them adapt as the disease progresses. Personal factors, which are defined as the particular background of a person's life and living which are not part of a health condition [5], can be important barriers and/or facilitators to this process, and rehabilitation often aims to enhance facilitating factors whilst underplaying the negative factors to achieve the most optimal functional and social reintegration outcomes. For example, education (for patients and families) is an integral part of MND management, but information provided must be appropriate to the patient's educational level; timing of end-of-life issues depend on a number of factors including coping skills, depression and anxiety, cultural issues, and functional status [6]; technological aids (which can vary considerably in cost) need to suit the patient's socioeconomic status, which impacts on their ability to fund these aids.

The International Classification of Functioning, Disability and Health (ICF) [5] aims to develop a common language for describing the impact of a disease at different levels. Within the ICF classification, MND-related impairments (muscle weakness) can limit “activity” (reduced mobility and self care) and “participation” (work, family, and social reintegration). The ICF also acknowledges that environmental factors (physical, social, and attitudinal environment in which people live and conduct their lives) and personal factors (intrinsic influences such as race, gender, and coping styles) interact with all the other constructs within the ICF to affect the person's overall experience of living with their condition. A set of relevant ICF categories in MND would be useful in both clinical and research settings given the rare incidence of MND and diverse and challenging nature of the symptoms. It has been also been highlighted that current outcome measures do not capture the entire spectrum of issues in MND [7]; use of the ICF categories could contribute towards development of appropriate outcome measures for MND. However, at present, personal factors are not classified in the current version of ICF, which represents a significant gap in the overall biopsychosocial view of the condition.

This preliminary study aims to identify the personal factors that are relevant in persons with MND from their perspective, in an Australian cohort.

## 2. Methods

### 2.1. Participants and Setting

A community-based MND group was recruited through a tertiary MND multidisciplinary clinic that services Victoria, Australia, including metropolitan and rural regions. Selection criteria included diagnosis of MND according to the El Escorial criteria [8] as diagnosed by a neurologist, residence in Victoria, community based (nonhospital inpatient), ability and willingness to give informed consent. Exclusion criteria included severe cognitive issues or dementia and other substantial medical, neurological, or psychiatric disorders. Screening of exclusion criteria was done by a neurologist through consultation of the medical records and liaison with the treating clinicians of the patients. This study was approved by the Melbourne Health and Calvary Healthcare Bethlehem Human Research and Ethics Committees.

All participants (*n* = 59) who met the criteria were contacted by mail and invited to participate in the study. Those who replied affirmatively (*n* = 44) were contacted by telephone by the primary author who explained the study further and organised an interview appointment. Participants were interviewed at a venue of their choice (half were interviewed at home or at hospital and half over the telephone) for one hour with rest breaks.

### 2.2. Questionnaires

Interviews commenced with an open-ended self-report questionnaire. Participants were asked, “What are the main problems you face in your everyday life? If possible, can you list and prioritize up to 10 issues that you feel are the most pressing problems you face in everyday life?” Participants were asked to include intrinsic factors that impacted on their experience of these problems, such as their ability to cope. Some who had difficulties with verbal communication chose to write their responses and/or use communication devices.

From the participant responses (from the open-ended questionnaire), all problems relating to personal factors (currently not coded within the ICF) were grouped under “personal factors” and categorized thematically under major headings, which included demographic factors (gender, race, age, and educational status), emotional states (depression, stress, anxiety, and fear), coping strategies and styles (problem-based coping and denial), personality, beliefs (includes self-efficacy, religious beliefs and values, personal and cultural), attitudes (of the patient), and “other” (perceived social support). Reports unrelated to personal factors have not been included in this paper as they are not the primary focus. 

Self-administered (patient) questionnaires followed the open-ended questionnaire:

- sociodemographic and medical status questionnaire,
- amyotrophic lateral sclerosis functional rating scale (ALSFRS) [9] to determine severity of MND. This is a 48-point measure of disability in MND with excellent validity and reliability and can be administered over the phone. It is determined by scoring 0–4 for each of the twelve domains (speech, salivation, swallowing, handwriting, cutting food and handling utensils, dressing and hygiene, turning in bed and adjusting bed clothes, walking, climbing stairs, dyspnoea, orthopnea, and respiratory insufficiency). A lower score indicates more disability,
- depression, anxiety, and stress scale (DASS) [10], a 21-item instrument with acceptable to excellent internal consistency and concurrent validity, consisting of three 7-item self-report scales designed to measure the negative emotional states of depression, anxiety, and stress. Participants rate the extent to which they experienced each state over the past week on a 4-point Likert rating scale,
- brief COPE [11], a coping inventory, with good reliability and validity, of 14 subscales (active coping, planning, positive reframing, acceptance, humour, religion, using emotional support, using instrumental support, self-distraction, denial, venting, substance use, behavioural disengagement, and self-blame) measuring responses relevant to effective and ineffective coping.

Assistance was provided where necessary by the interviewer and all patient responses were clarified where possible with their caregivers.

### 2.3. Statistical Analysis

Results were described by mean and standard deviation (SD) for continuous nonskewed data and as frequency (%) for categorical data. Each identified personal factor was listed once regardless of the frequency of identification either by a single or multiple participant(s) for simplicity.

## 3. Results

Mean age of participants was 61 years (SD 9.8), and male: female ratio was 3 : 2. Mean time since diagnosis was 3.6 years. Half (*n* = 18, 41%) of the participants had severe disease as classified by ALSFRS (ALSFRS 0–24) (see Table 1). Participants appeared grossly cognitively intact based on simple observation during the interviews. Personal factors identified that were relevant in persons with MND to the experience of their condition are listed in Table 2. Some factors crossed over two categories. For example, “self-esteem” encompassed both a belief (that the patient was worthy) and an emotion (of pride) and is, therefore, listed under both categories. A significant proportion of the participants were depressed (*n* = 19, 43%), anxious (18, 41%) and/or stressed (11, 25%) and problem focused coping strategies were used much more commonly than emotion-focused coping strategies (see Table 3).

## 4. Discussion

This is the first study that has identified personal factors that shape a person with MND's experience of their condition. These factors include demographic factors (gender, race, age, educational status, and socioeconomic status), emotional states (depression, stress, anxiety, and fear), coping strategies and styles (problem-based coping and denial), personality, beliefs (includes self-efficacy, religious beliefs, and personal values), attitudes (of the patient) and “other” (perceived social support). Rates of depression and anxiety were high and a broad range of coping strategies were used although problem-focused coping strategies were preferred. The mean participant age, gender, time since diagnosis, distribution of type, and severity of disease (based on ALSFRS-R) were similar to those reported by others [12]. The participants represented a broad range of disability and disease severity, with demographic and diagnostic characteristics typical of MND.

These findings (of depression, anxiety, and a range of coping strategies) and the identified personal factors are consistent with other reports in MND literature. Rates of depression and anxiety are reported to be 0–44% and 0–30%, respectively, in persons with MND [13]. This is not surprising given the progressive and fatal nature of their disease. However, it is also generally thought that despite disease progression, most MND patients adjust effectively to their illness and in fact are often perceived to be particularly positive people [14]. The impact of personal factors on the lived experience and QoL of MND should not be underestimated. It has been previously reported that QoL appears to be more dependent on “psychological and existential issues, social support and spirituality” rather than physical factors [15]. These findings were supported by Chiò et al. [16] who found that the main determinants of quality of life in MND were social support, depression, religiosity, and socioeconomic status. More recently, Roach et al. concurred that it was likely that characteristics such as personality, social relationships, and spirituality could be more important for QoL [17] than progression of the disease per se. The importance of coping strategies in the experience of MND is further supported by Gallagher and Monroe [18] who surmised that MND is not a static disease but a progressive disorder that required different coping strategies at different stages of the disease. Matuz et al. [14] also found that the best predictors for the severity of depressive symptoms in MND were perceived social support (especially a supportive marital relationship) and coping potential (information seeking and strategies of emotional avoidance behaviour). For example, a combination of confronting and avoiding coping strategies might be useful for MND patients, because search of information and support may help them to initiate actions that ensure optimal future care [14]. On the other hand, emotional avoidance behaviour (e.g., choosing isolation or denial) could protect them from psychological distress and despair [19]. However, as the disease progresses, avoidance is no longer an adaptive strategy, as it prevents patients from taking appropriate measure to cope further with the illness [14]. 

Personality appears to be another personal factor that plays a significant role in the experience of MND. It has been found that MND patients (*n* = 31) who scored higher on the agreeableness personality dimension had higher QoL initially, but the reduction of QoL over 12 months was significantly steeper than in patients who scored lower on agreeableness, suggesting that being less agreeable might serve as a protective factor with respect to QoL [20]. Nelson et al. [21] reported that personality traits such as optimism, flexibility, and humor were important in coping. As for beliefs, it has been reported that the belief that fate controls one's health and a person's belief in “powerful others” such as doctors changes with disease progression in MND [22]. Interestingly, no cultural beliefs were mentioned in this cohort, possibly because of the homogeneity in race (100% Caucasian) in this cohort, but religious beliefs were frequently mentioned, consistent with the high proportion (64%) of Christianity practiced in Australia [23] and with previous reports that religion is one of the most important coping mechanisms in a fatal disease such as MND [24]. Finally, other studies have reported that the most important personal values in MND patients were benevolence, self-direction, and universalism [25]. These values were not specifically explored in this cohort.

Under the UK Department of Health's National Service Framework for Long-term Neurological Conditions [26], MND is a “progressive condition” with a rapid deteriorating course. Multiple sclerosis is another long-term progressive neurological condition but with a more “intermittent,” slowly “progressive” or “stable” nature. Comparison of relevant personal factors in MND with other “long-term neurological conditions” is challenging as it differs from other conditions in that there is constant deterioration and, therefore, ongoing “change and adaptation” [27]. It is also unique in that given the limited life span, changes in personal factors can be studied through the entire spectrum of the disease, as described above. There are few other reports relating to personal factors in other long-term neurological conditions. In identifying the ICF core set for multiple sclerosis, Khan and Pallant [28] suggested ten categories which included socioeconomic status, coping ability, attitudes and patient beliefs, self-efficacy, dependence on others, mood and affect, heat intolerance, fatigue, personality and temperament, and patient attitude towards the biopsychosocial impact of multiple sclerosis. Many of these are comparable with this MND cohort. Other reports have shown that in both MND and multiple sclerosis, psychological adaptation to deteriorating function is an important factor in perceived QoL and emotional well-being [29]. The impact of personal factors on participation in the rehabilitative process in other neurological conditions has also been emphasized—a recent study showed that rehabilitation professionals attempting to engage people with multiple sclerosis in a physical activity programme needed to consider adopting an individualised approach to barrier management which takes into account personal beliefs and perceptions regarding physical activity engagement [30]. Hence, despite some of the unique features of MND, many of the findings in this study are likely to be relevant to other neurological conditions.

MND takes its toll on the patient and family especially as the disease progresses and loss of independence occurs. Understanding the personal factors involved helps with the palliative rehabilitation process. As part of this process, advice with regards to adequate coping strategies and provision of an adequate amount of disease- and support-related information at any one time and encouraging patients to seek social support [14] is crucial. Hence, referrals to support groups and counselling and education of patients and their families (often their caregivers) are important. Subgroups of patients who are more likely to adapt poorly to a new diagnosis of MND can also be identified early with explorative interviews that specifically target coping, depression, anxiety, social withdrawal, and quality of life [31]. Targeted earlier intervention can be provided for this subgroup. Frank discussions facilitate understanding of the disease and improve coping skills. Referrals to the local MND associations are also recommended as these provide patients and families with ongoing support, resources and equipment needs. Psychotherapy should also be considered to assist with coping strategies [14] and antidepressants may be used. Anxiety is difficult to measure due to physical confounding symptoms such as shortness of breath, muscle cramps, and restlessness. Anxiety can be treated with psychotherapy and training in relaxation and breathing techniques as well as participation in support groups. With good support, mental health and quality of life can remain stable despite deteriorating physical health [32]. In addition, an often-neglected part of rehabilitation in MND is support for continuation of work [33]. Understanding personal factors can help target the subgroup of MND patients who wish to continue work—it has been found that intrinsic reasons (motivation resulting from a person's interest in and enjoyment of the work), followed by age, disability severity, and accessibility of travel [34] are the strongest predictors for this group.

The limitations in this study include cross-sectional survey (no longitudinal information), highly selective cohort (all already receiving tertiary multidisciplinary care, and willing research participants). Interviews were challenging given the fragile emotional and physical status of the participants. Self-reported information was used and validated as best with caregiver and medical records. The cohort covers a wide geographical population in Victoria and is representative of the wider sample of pwMND. 

MND is a devastating illness for patients and families. Palliative care and rehabilitation has much to offer this population. For improved consensus of care and communication amongst treating clinicians, the framework of ICF should be explored in this population and further expanded to take into consideration individual personal factors which impact significantly upon the experience of illness and the rehabilitation process. This preliminary study identifies intrinsic factors reported by patients with MND and may be the first step in the development of personal factors within the ICF classification.

## Figures and Tables

**Table 1:** Characteristics of motor neurone disease/amyotrophic lateral sclerosis (MND/ALS) participants—demographics and disease features.

Variable	Average/Frequency
Age (mean ± SD (range)	61 ± 9.8 (43–80)
Sex [*n* (%)]	
Male	29 (65.9)
Female	15 (34.1)
Marital status (*n*, %)	
Married/partner	34 (77.3)
Divorced/separated/single	10 (22.7)
Race (*n*, %)	
Caucasian	44 (100%)
Living [*n* (%)]	
Alone	8 (18.2)
Family	36 (82.8)
Geographical area	
Metropolitan [*n* (%)]	27(61.4)
Rural [*n* (%)]	17 (38.6)
Diagnosis of ALS (El Escorial criteria) [*n* (%)]	
Clinically definite ALS	14 (31.8)
Clinical probable ALS	17 (38.6)
Probable ALS (Lab Supported)	5 (11.3)
Possible ALS	4 (9.1)
Suspected ALS	4 (9.1)
Amyotrophic lateral sclerosis functional rating scale-revised (ALSFRS—R [*n* (%)]	
0–12	3 (6.8)
13–24	15 (34.1)
25–36	12 (27.3)
37–48	14 (31.8)
Comorbidities (*n*, %)	
Yes	28 (63.6)
None	16 (36.4)
≥2 comorbidities	14 (50.0)
Clinical symptoms (*n*, %)	
Fatigue	34 (77.3)
Pain	22 (50.0)
Spasticity/cramps/spasms	32 (72.7)
Emotional lability	21 (47.7)
Shortness of breath	8 (18)

**Table 2:** Personal factors reported by participants with motor neurone disease (MND) which affected their experience of living with MND.

Personal factors category	Personal factors examples
Demographic factors	Gender
	Race
	Age
	Educational status
	Socioeconomic status

Emotional states	Frustration
	Depression
	Stress
	Anxiety
	Fear
	Worry
	Degrading
	Grumpy
	Loss of confidence
	Anger
	Self-esteem
	Embarrassment
	Hope (both hopeful and hopeless)
	Guilt
	Grief
	Loss
	Gratitude

Coping strategies and styles	Problem solving
	Search for information
	Planning
	PositivityAcceptance
	Humour
	Religion
	Using support
	Denial
	Avoidance

Personality	Stubborn
	Easy-going

Beliefs	Religious beliefs
	Self-esteem

Attitudes (of the patient)	Grateful attitude (towards family and health professionals)
	Fighting attitude
	Attitude towards assisted suicide
	Being organised

“Other”	Perceived support

**Table 3:** Results of depression, anxiety, and stress scale (DASS) and brief COPE.

DASS	[*n*, (%)]	
*DASS Depression*		
Normal	25 (56.8%)	
Mild	9 (20.5)	
Moderate	4 (9.1%)	
Severe	3 (6.8%)	
Extreme	3 (6.8%)	
*DASS Anxiety*		
Normal	26 (59.1%)	
Mild	6 (13.6%)	
Moderate	4 (9.1%)	
Severe	4 (9.1%)	
Extreme	4 (9.1%)	
*DASS Stress*		
Normal	33 (75.0%)	
Mild	6 (13.6%)	
Moderate	2 (4.5%)	
Severe	3 (6.8%)	
Extreme	0	

Brief COPE variables	Mean (SD)	Range

*Problem focused coping strategies*		
Active coping (2–8)	6.3 (1.6)	2–8
Planning (2–8)	6.2 (1.7)	2–8
Positive reframing (2–8)	6.0 (1.8)	2–8
Acceptance (2–8)	7.4 (1.1)	2–8
Humour (2–8)	5.0 (2.4)	2–8
Religion (2–8)	4.0 (2.3)	2–8
Using emotional support (2–8)	6.3 (1.5)	3–8
Using instrumental support (2–8)	5.7 (1.7)	2–8
*Emotion-focused coping strategies*		
Self-distraction (2–8)	5.7 (2.0)	2–8
Denial (2–8)	2.9 (1.4)	2–8
Venting (2–8)	3.6 (1.6)	2–8
Substance use (2–8)	2.7 (1.5)	2–8
Behavioural disengagement (2–8)	2.8 (1.4)	2–7
Self-blame (2–8)	2.7 (1.0)	2–6
